# Spatial position constraint for unsupervised learning of speech representations

**DOI:** 10.7717/peerj-cs.650

**Published:** 2021-07-21

**Authors:** Mohammad Ali Humayun, Hayati Yassin, Pg Emeroylariffion Abas

**Affiliations:** Faculty of Integrated Technologies, Universiti Brunei Darussalam, Jalan Tungku Link, Brunei

**Keywords:** Low resource speech, Representation learning, Multitasking, Geometric constraint

## Abstract

The success of supervised learning techniques for automatic speech processing does not always extend to problems with limited annotated speech. Unsupervised representation learning aims at utilizing unlabelled data to learn a transformation that makes speech easily distinguishable for classification tasks, whereby deep auto-encoder variants have been most successful in finding such representations. This paper proposes a novel mechanism to incorporate geometric position of speech samples within the global structure of an unlabelled feature set. Regression to the geometric position is also added as an additional constraint for the representation learning auto-encoder. The representation learnt by the proposed model has been evaluated over a supervised classification task for limited vocabulary keyword spotting, with the proposed representation outperforming the commonly used cepstral features by about 9% in terms of classification accuracy, despite using a limited amount of labels during supervision. Furthermore, a small keyword dataset has been collected for Kadazan, an indigenous, low-resourced Southeast Asian language. Analysis for the Kadazan dataset also confirms the superiority of the proposed representation for limited annotation. The results are significant as they confirm that the proposed method can learn unsupervised speech representations effectively for classification tasks with scarce labelled data.

## Introduction

Despite the impressive performance of supervised deep learning models for Automatic Speech Recognition (ASR), these models require a huge amount of manually labelled data for training (Chiu et al., 2018). This dependency on annotated labelled data reduces the effectiveness of supervised models for low resource languages. Languages with a limited amount of annotated speech are considered to be low resource languages. Limited availability of annotated speech may be due to a lack of linguistic expertise and resources required to label and maintain large transcribed datasets. Recently, unsupervised learning for speech processing tasks has received growing interest from researchers and industries. Unsupervised techniques are capable of learning useful patterns from speech without annotations, which are relatively easier to obtain as compared to annotated and labelled speech. Generally, unsupervised speech processing can be categorized into two main directions, as presented by the zero resource speech challenges (Dunbar et al., 2018; Dunbar et al., 2019). One of the research directions is to find similar segments of repeating patterns in speech for the clustering of relevant phones or words, whilst the other more popular approach is of learning a transformation from speech, into a more useful representation. Traditionally, speech is transformed into spectral or cepstral features and fed as input to classification models. On the other hand, unsupervised representation learning models use statistics of the dataset to find a compact representation with minimum correlation among the dimensions whilst retaining only critical information to be fed as input to the classification models. Naturally, unsupervised representations commonly outperform the traditional features for low-resourced annotation settings (Ravanelli et al., 2020).

One of the most popular approaches for projecting unlabelled data to an embedding in lower dimensions is via an auto-encoder (Bengio, Courville & Vincent, 2013). Auto-encoders are specialized unsupervised neural network architectures that learn to transform data into a lower-dimensional representation. A typical auto-encoder is composed of similar input and output layers that are connected via a low-capacity bottleneck layer within the intermediate layers to capture a low dimensional representation. This architecture allows the network to produce a lower-dimensional representation that has minimum correlation while discarding redundant information. Apart from the lower capacity of the intermediate layers, additional constraints during training can also be added to allow the network to learn more complex features from the data.

Some of the recent studies such as (Kamper, 2019) and (Bastiaan Kleijn et al., 2019) have proposed incorporating the geometric structure of a dataset as an additional constraint during network training. There are two popular approaches that have been applied for utilizing the geometric structure of data. The first approach relies on utilizing geometrically adjacent data points as input–output pairs for auto-encoder training (Kamper, 2019), whilst the second approach works by forcing the transformed representations from the cloned networks to reflect the original distance between the points sampled randomly as inputs for the cloned networks (Bastiaan Kleijn et al., 2019). The former approach requires distance matrix computation over the complete dataset to find the most similar data points. However, distance computation for all possible combinations in a huge dataset is naturally a resource-intensive exercise. On the other hand, the latter relies on random sampling from input space for distance projection in the representation, which may not guarantee complete description for data.

This paper proposes a novel unsupervised representation learning technique, by considering the geometric structure of the data. In contrast to other metric-learning methods, the technique proposed in this paper does not require random sampling or complete distance matrix computation. Instead, the proposed architecture uses regression to the geometric position for each data point as a constraint, where the position of each point is estimated by its cosine distance from an arbitrary reference. It has been shown that the auto-encoder model with the proposed constraint achieves improved performance as compared to the benchmark handcrafted speech features for speech classification. Automatic Key-Word-Spotting (KWS) has been used as the classification task to evaluate the proposed method. KWS refers to the recognition of small vocabulary, isolated words by a lightweight classifier, capable of being run locally on handheld devices. Existing models have mostly considered KWS as a supervised task, which limits their application to high-resource languages. Consequently, this study has analyzed KWS for limited annotation settings relevant for low-resource languages. The proposed model has been tested using different languages for unsupervised training and supervised evaluation. This includes the English language as the zero-resource challenge proposals have mostly used English datasets, with and without labels, for its supervised evaluation as well as unsupervised learning (CoML_Team, 2021). For the evaluation of generalization across languages, a new dataset has been collected for spoken digits in ‘Kadazan’. Kadazan is an indigenous Southeast Asian language considered as an endangered language due to its limited linguistic resources.

The research questions can be summarized as follows:

• Can the proposed geometry-based auto-encoder, trained on unlabeled speech, learn to extract speech features which are useful for the keyword spotting task?
• How is the performance of the proposed method as compared to the traditional speech cepstral features?
• Is the proposed method applicable for a low-resource language, such as the Kadazan language?

The rest of the paper is organized as follows: ‘Literature Review’ highlights recent trends in unsupervised deep representation learning as well as models particularly focused on speech. The ‘Method’ section describes the proposed multi-task model with positional constraint, with the section also listing the parameters used for the proposed neural network architecture. ‘Experiments and Results’ provides the experimental setup and evaluation results for the proposed model, and finally, ‘Conclusions’ concludes the paper.

## Literature Review

This section reviews recent literature related to unsupervised representation learning. The ‘Unsupervised representation learning’ section presents a survey on unsupervised representation learning in general and ‘Representation learning for speech’ summarizes representation learning models that have been proposed specifically for speech. Finally, ‘Geometric distance in speech representation learning’ focuses on geometric structure-based representation learning models for speech that are most relevant to the model proposed in this paper.

### Unsupervised representation learning

Some of the traditional techniques for transformation of data to lower dimensions are Principal Component Analysis (PCA), factor analysis, sparse coding, random projection, multidimensional scaling, etc. (Tipping & Bishop, 1999). These transformations decompose data into components based on fixed statistical or geometric criteria. PCA and factor analysis aim to find a projection that maximizes variance across data points and among the target dimensions, respectively (Ramashini et al., 2019). On the other hand, sparse coding utilizes and learns a dictionary, such that input data can be decomposed into a linear combination of a sparse code and the learned dictionary themselves (Lee et al., 2007). When projecting to a lower number of dimensions, random projection and multidimensional scaling aim at preserving geometric structure within data points (Dasgupta & Freund, 2008).

All of these transformations may easily be achieved by using specialized auto-encoders (Ghahramani & Roweis, 1999). Auto-encoders have an encoder that maps the input to the representation and a decoder that reconstructs the input from the representation. Restrictions or constraints, imposed on the representation layer of the auto-encoder, help to extract components of interest from the input data, with the size of the representation layer being the most common restriction. The number of units in the representation layer is usually lower than the number of input and output layer units to capture salient components of data, which are critical for reconstruction. Representation layer weights and outputs can also be penalized by adding a term for their norms to the training objective function to keep the values in a lower range or to encourage sparse activations. Another popular approach used to retain salient information in the representation is de-noising. With de-noising, input data is augmented during training with manually corrupted data whilst keeping the target output clean so that the representation learned is invariant to noise. A similar effect can also be achieved by penalizing the representation layer outputs for variation in input, which is referred to as the contractive loss penalty (Rifai et al., 2011). Furthermore, self-supervised training has been applied effectively for representation learning (Raina et al., 2007). In a self-supervised setting, the auto-encoder is trained to map input data to its transformed version at the output, with the transformed output computed through manual processing techniques over the input data. Another unsupervised technique is multitask learning, i.e., sharing the representation across multiple tasks such that the model is trained to simultaneously reconstruct multiple outputs whilst sharing a common bottleneck representation (Maurer, Pontil & Romera-Paredes, 2016). Multitask-learning forces the representation to capture key information relevant to all outputs.

Auto-encoders can also be adapted for manifold learning by adding a compulsion on the representation to preserve geometric distance between data points from the input space. Neural networks incorporating spatial structure from the input data by considering geometric distance metrics are also called deep metric learning networks. Multiple cloned neural networks, named Siamese or Triamese networks (Sohn, 2016), have been used to preserve the distance between data points selected from the input space. The geometric distance between output representations of the cloned networks fed with sampled data points is trained to be proportionate to the original distance from the input space.

### Representation learning for speech

Variants of auto-encoder based architectures have been employed to capture a representation for speech that makes spoken words easily distinguishable for classification. Amongst manually extracted speech features, Mel Frequency Cepstral Coefficients (MFCC) have remained the most effective for speech classification tasks. Filter banks are applied to the Mel-scaled spectra from short quasi-stationary time windows, with coefficients of the Discrete Cosine Transform (DCT) for the sequence of logarithmic filter bank energies, forming the MFCC vectors for each time window (Sahidullah & Saha, 2012). MFCC coefficients have little correlation, which make them suitable as input for classification models. Representation learning models attempt to find a transformation for speech that is better than MFCC.

Auto-encoder based architectures trained on speech features are focused on extracting a representation that retains critical information, whilst discarding unimportant information such as speaker and environmental characteristics. After training, the encoder may be used to transform unseen speech into its suitable representation (Renshaw et al., 2015). Some specialized representation learning frameworks that have been proposed for speech are based on Variational Auto-Encoder (VAE), adversarial networks, self-supervised learning, autoregressive prediction, and self-supervision (Goodfellow et al., 2020; Gregor et al., 2014; Kingma & Welling, 2019). Different variants of VAE are designed to learn stochastic, quantized, or factorized representation for the segregation of phonetic information (Chorowski etal, 2019; Feng & Lee, 2019; Kingma & Welling, 2019). Adversarial learning based models are designed to deceive a classifier for features in speech irrelevant for ASR (Ganin et al., 2017; Meng et al., 2018), whilst autoregressive models are trained to predict speech features ahead in time as output targets (Chung et al., 2019; Oord etal , 2018). On the other hand, models based on self-supervision generally attempt to reconstruct pre-computed speech features using raw speech as input (Pascual et al., 2019; Ravanelli et al., 2020).

### Geometric distance in speech representation learning

Multiple variants of auto-encoder have incorporated the geometric structure of speech samples for finding a suitable representation. Similarly, this paper also focuses on geometric structure as an additional constraint for the Auto-encoders. Models proposed in recent literature, which utilize the dataset geometry, are mostly based on either cloned networks or the Correspondence Auto-Encoder (CAE) (Bastiaan Kleijn et al., 2019; Kamper, 2019). CAE refers to an auto-encoder model that utilizes similar data points as corresponding input–output pairs, with these points grouped as pairs based on geometric distances within the input dataset (Jansen & Van Durme, 2011; Kamper, 2019). On the other hand, cloned neural network models such as Siamese or Triamese networks, process pairs or triplets of input data points in parallel by considering their distance (Bastiaan Kleijn et al., 2019; Riad et al., 2018). In the Siamese networks, two data points are sampled and are fed to two different neural networks with tied weights. The training objective for both networks is to minimize the geometric distance between representations for close-by points while maximizing the distance for faraway points. In contrast to the Siamese networks, the Triamese networks sample three points simultaneously to minimize the triplet loss, with one data point selected as an anchor along with one adjacent and one distant point. The anchor and two references are then fed to the three cloned networks. Distance between output representations for the anchor and its nearby point is trained to be minimized whilst the distance between representations for the anchor and the distant point is maximized. Both CAE and Triamese networks have also been proposed to complement one another as a joint model, referred to as Correspondence Triamese Auto-Encoder (CTAE) (Last, Engelbrecht & Kamper, 2020). CTAE learns to minimize triplet loss in the representation layer whilst using the two similar points from the triplet as input–output pair for reconstruction. The combination of correspondence and triplet loss i.e., the CTAE, has been shown to be better than both CAE and Triamese networks individually, confirming that both capture different complementary information, which results in improved performance for CTAE.

However, all these techniques either compute the distance matrix for the complete dataset, which can be computationally complex, or rely on random sampling to estimate geometric distances. CAE relies on distance measure approximation for all possible combinations from the input dataset, which may require a substantial amount of resources, especially for a large dataset. On the other hand, cloned networks sample associated points for each input to reflect the distance in the representations (Kamper, 2019; Sohn, 2016) and as such, the performance of the model is significantly influenced by sampling strategies employed for selecting associated points.

## Method

This paper proposes a spatial constraint-based representation learning model that does not require complete distance matrix computation or random data sampling for training. Section ‘Spatial position constraint’ explains the proposed method for the calculation of spatial or geometrical positions for data points as a scalar value. Regression to the position scalar is used as the secondary task for the auto-encoder. Consequently, ‘Auto-encoder architecture’ describes the architecture for the auto-encoder with the position used as a constraint.

### Spatial position constraint

The spatial constraint is based on the global position of each point within the input data space represented by the cosine distance of that point from an anchor. The mean of the speech feature set is taken as the anchor.

MFCC vectors are taken as input speech features of the multitasking auto-encoder. These features are then transformed into the representation, which is shared by the two parallel outputs: the primary and secondary output decoders. The primary output decoder reconstructs the MFCC features whilst the secondary output decoder learns to generate the position of each speech sample, with the positions determined from Mel scaled spectrogram-based features. It is noted that the spectrogram frequencies are mapped to the human-inspired Mel scale to emphasize the lower frequency range of human speech (Stevens, Volkmann & Newman, 1937). A mean vector containing arithmetic averages of components in the feature vectors across the complete sample set serves as an anchor to compute relative positions of all points. The position of each sample is then taken as the cosine-distance between its Mel-spectrogram and the mean of all Mel-spectrograms in the dataset.

**Figure 1:**
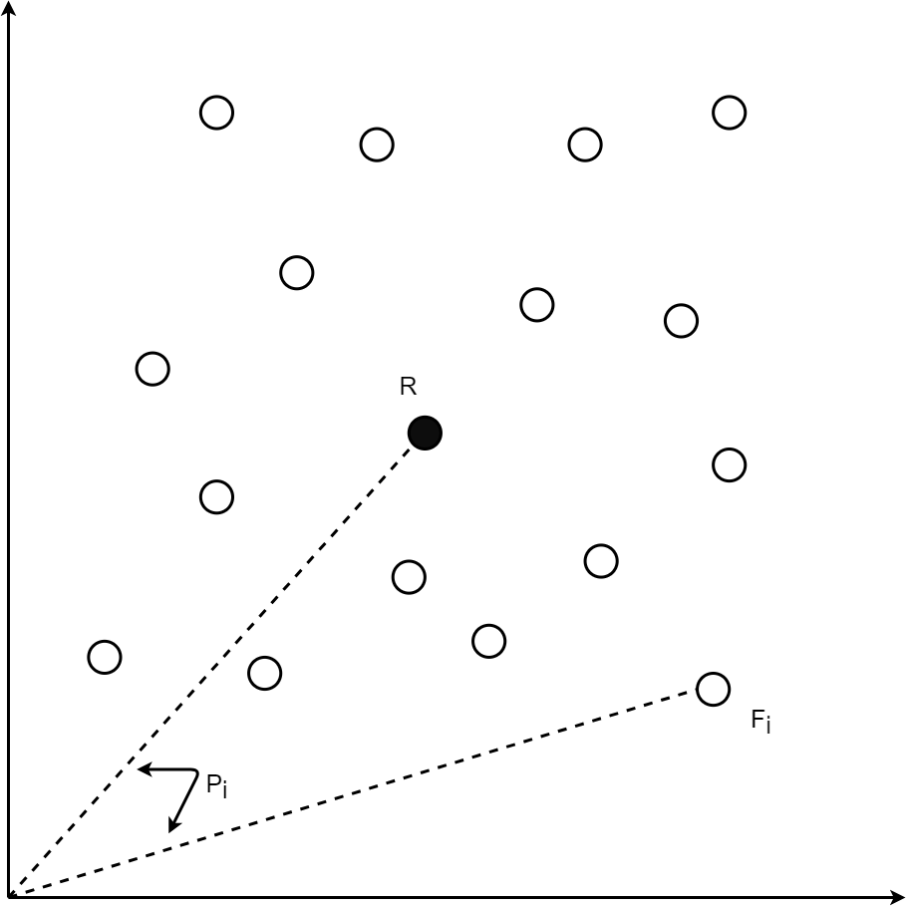
Position for data point *i* having a feature vector *F*_*i*_.

For each speech sample *i*, the Mel-spectrogram based feature vector *F*_*i*_ may be obtained by ravelling its Mel-spectrogram into a single axis by row-major order. Reference anchor point vector *R* for the dataset may be taken as the means of all components in the feature vectors. Given the presence of *m* points for speech sample *i*, the *j*^*th*^ component of the anchor vector *R* i.e.,  *r*_*j*_, is given by (1). The spatial position scalar *p*_*i*_ for every point *i* is the cosine distance of its feature vector *F*_*i*_ from the reference vector *R* where cosine distance is obtained by normalizing the dot product of both vectors by the product of their L2 norms as given by (2). The position *p*_*i*_ for speech sample *i* is used as secondary output in the auto-encoder.

(1) \begin{eqnarray*}{r}_{j}& = \frac{\sum _{i=0}^{m}{f}_{ij}}{m} \end{eqnarray*}

(2) \begin{eqnarray*}{p}_{i}& = \frac{\sum _{j=0}^{n}{r}_{j}{f}_{ij}}{\sqrt{R{R}^{T}}\sqrt{{F}_{i}{F}_{i}^{T}}} \end{eqnarray*}

During training, the auto-encoder learns to generate both the MFCC vector as well as the position *p*_*i*_, for every input. Figure 1 depicts the position computation for data point *i* with feature vector *F*_*i*_. Spectrograms for the position calculation are obtained by Fast Fourier Transform with 2048 linear frequency bins mapped to 128 Mel-scale chunks for each 25 ms time window. Various metrics including cosine, correlation, L1, and L2 distances have been analysed for position calculation, with cosine distance found to be the best and hence, has been chosen for the position calculation.

**Figure 2:**
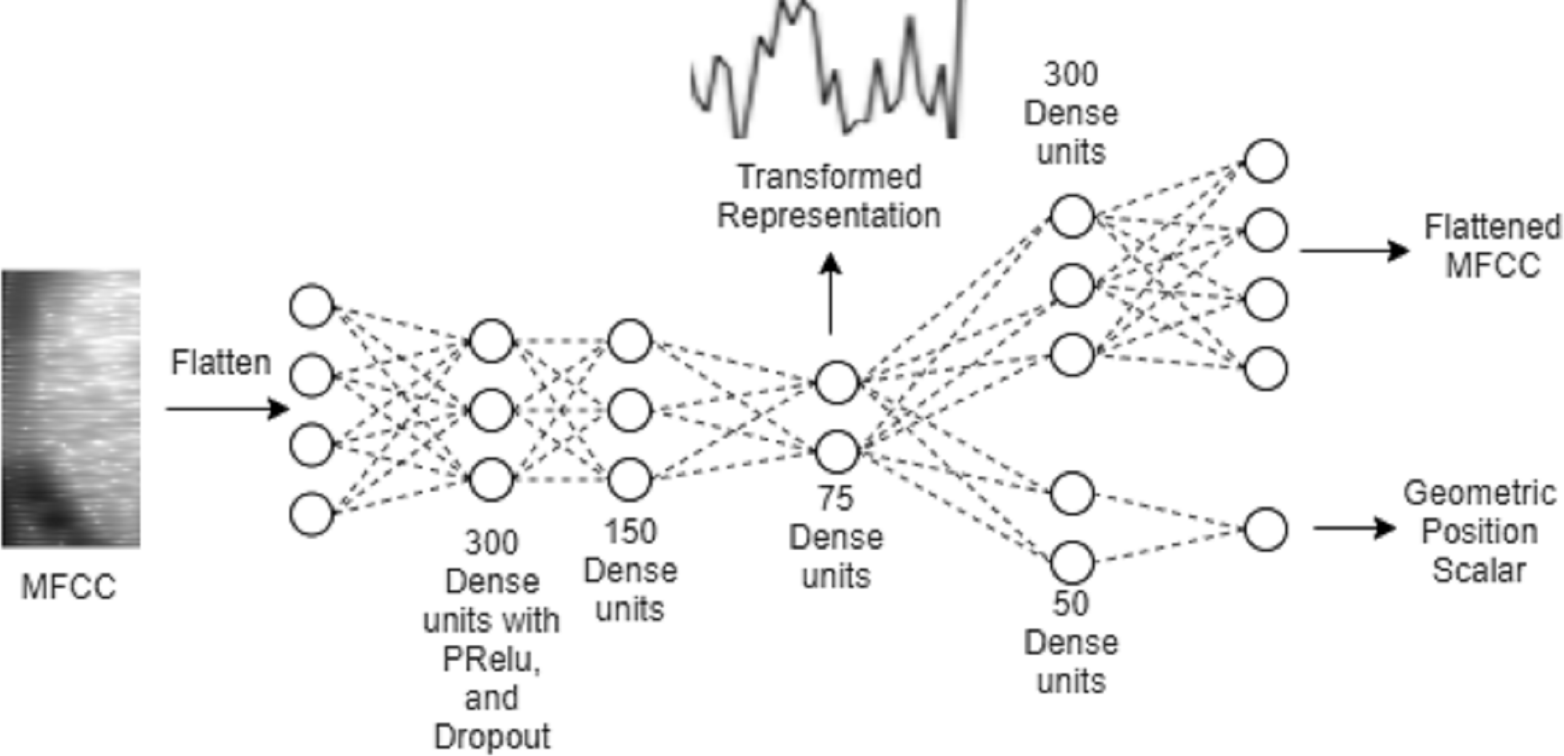
The proposed auto-encoder model.

### Auto-encoder architecture

Figure 2 illustrates the architecture of the proposed multitasking auto-encoder model. The encoder maps MFCC features from input to the representation, which is then used to reconstruct both, input MFCC features as well as the geometric position. A common encoder is shared by the two decoder networks.

The auto-encoder architecture can be divided into three different parts: (1) the encoder, which learns the representation, (2) the primary decoder, which reconstructs the flattened MFCC, and (3) the secondary decoder, which predicts the geometric position. Three hidden layers following the input layer constitute the encoder. The first and second hidden layers comprise of 300 and 150 fully connected units, respectively, with PRELU activations, dropout, and batch normalization. The third hidden layer is the representation layer, which is used to capture the representation. This representation layer consists of 75 units and batch normalization without any activation function.

The primary decoder has a single hidden layer followed by the output layer for MFCC reconstruction, with the hidden layer composed of 300 units without any activation function. This is to keep the decoder simpler so that more information is captured in the encoder. The secondary decoder also has a single hidden layer but consisting of only 50 units with PRELU activations and batch normalization. Output unit of the secondary decoder gives prediction of the scalar value representing the geometric position.

Training of the auto-encoder is based on optimizing mean square error for outputs of both decoders. For the auto-encoder input and primary output, all the speech samples are transformed to 20 MFCC coefficients, which are then reshaped to linear vectors, with time axis indices changing faster than the cepstral coefficients.

## Experiments and Results

The representations learnt by unsupervised models are commonly evaluated by using them as input for a supervised classifier and then, comparing the outcomes with the traditional speech features. Performance of the supervised classification determines the ability of the unsupervised model to highlight information from the speech which is useful for classifications. In this paper, the representation learning model is trained on unlabelled speech whilst a labelled dataset is used for representation transformation by the trained encoder for evaluation. The following experiments and results aim to analyze the extent of accuracy improvement for speech classification by the proposed unsupervised representation over hand-crafted features in limited annotation settings.

### Dataset and experimental setup

The proposed model is evaluated using the Speech Commands dataset by Google Artificial Intelligence Yourself (AIY) Projects (Warden, 2018). This dataset consists of 64,727 audio files which are composed of 30 unique English words spoken by crowd-sourced speakers and is organized with speaker names. This dataset was originally developed for Key-Word-Spotting (KWS) research with an aim of designing light discriminator models that can run locally on low resource handheld devices to recognize certain keywords, which have been reserved to demand certain actions. Three partitions are made from the dataset: training, validation, and testing partitions. Each partition comprises of different sets of speakers without overlap. 6,799 and 6,836 speech samples are reserved for validation and testing, respectively. The dataset is a publicly available dataset (Warden, 2021). Labels for the dataset in the training partition have been ignored in this work, which allows the original training set to be considered as unannotated speech.

Retaining only limited labelled data for evaluation by a supervised classifier emulates the primary limitation of low resource languages, which commonly have a small amount of labelled training data available. Of course, unsupervised representation learning models would still need a large amount of unlabelled data. However, obtaining unlabelled data for unsupervised training is comparatively easier as it only needs recording of spontaneous speech for long durations rather than the challenging and troublesome task of manually annotating large datasets of low resource language for the purpose of supervised learning. Many works in the recent literature have used this technique of using unlabelled datasets of the English language to mimic low resource language for the purpose of evaluating the performance of unsupervised representation learning models (Chorowski etal, 2019; Kamper, 2019; Pascual et al., 2019). Furthermore, using the English language for the representation learning models has the added advantage of convenient comparisons with benchmark results (Chorowski etal, 2019; Kamper, 2019; Pascual et al., 2019).

**Figure 3:**
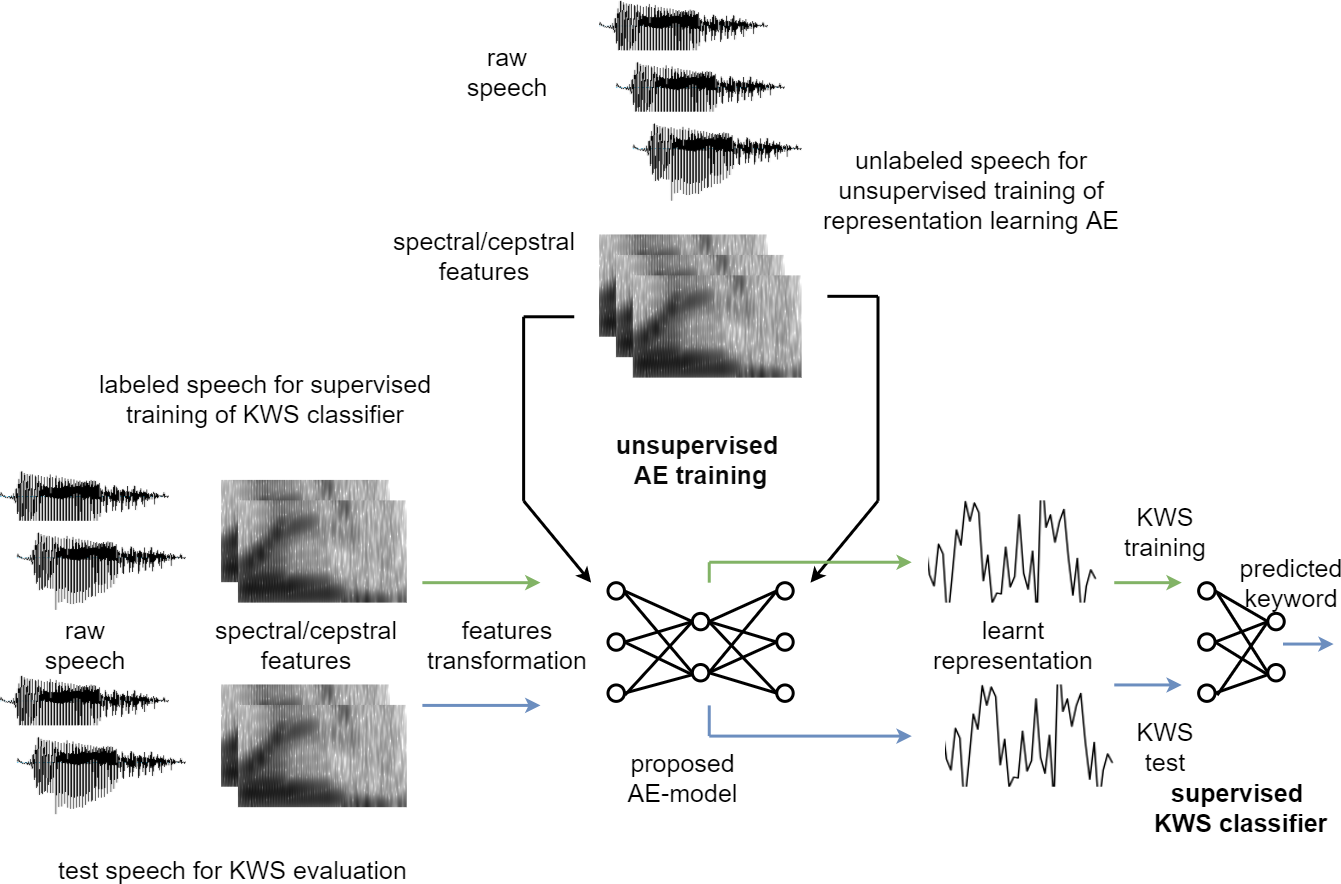
Experimental setup.

**Figure 4:**
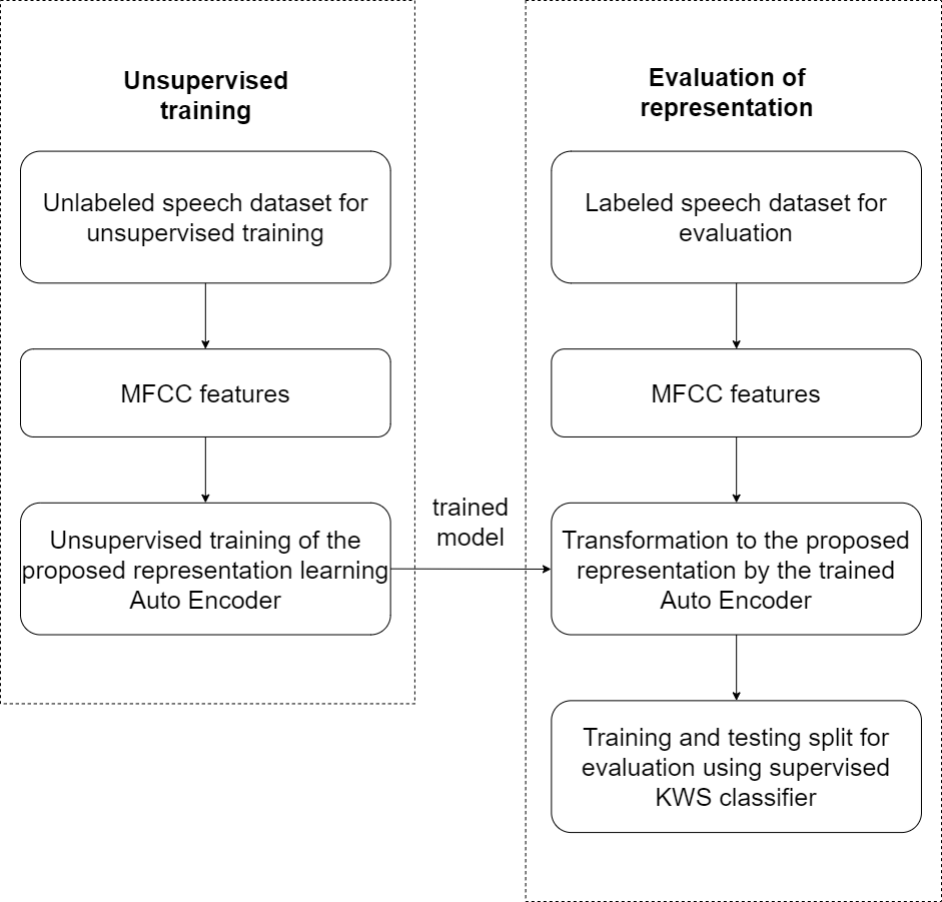
Experimental flow chart.

Figure 3 illustrates the experimental setup used to evaluate the effectiveness of the proposed unsupervised model. Breakdown of the experimental steps has also been presented as a flowchart in Fig. 4. The large unlabelled dataset amounting to 12,000 unannotated samples from the training partition is used to train the proposed auto-encoder based model, with MFCC features of the unlabelled dataset used for the unsupervised training.

After the unsupervised training of the auto-encoder based model, the MFCC features for a smaller labelled dataset are then transformed to the new representation by using the trained auto-encoder model. Only 3,000 labelled samples from the validation set, with new representation for the speech using the auto-encoder model, are then used to train the supervised feed-forward classifier for Key-Word-Spotting (KWS) evaluation. Using a relatively small number of labelled samples emulates a low resource language. Another 3,000 samples from the validation partition dataset are reserved for cross-validation based early-stopping of the supervised classifier. This feed-forward network is henceforth referred to as the Deep Neural Network (DNN) classifier for the analysis of results. The DNN is composed of a single hidden layer with PReLU activation functions, batch normalization, and dropout as well as an output layer based on Softmax.

Consequently, the test partition dataset with over 6,000 samples is also transformed by the trained unsupervised auto-encoder model to the new feature representation and is used to compute classification performance of the DNN. Source code for the proposed model is shared online (Humayun & Abas, 2021b).

Classification metrics by the DNN using the proposed representation as input are compared over the same dataset with a benchmark CNN for the particular task using the traditional speech features, i.e., MFCC, as in reference (Choi et al., 2019). The corpus paper has presented a baseline accuracy of 88.2% as a benchmark using 2D CNN architecture over spectrogram, designed specifically for low footprint KWS tasks (Sainath & Parada, 2015). Cho et al. (Choi et al., 2019) have also used the same dataset and reported up to 96.6% accuracy using temporal convolution over MFCC, however, the complete dataset has been used in the study, i.e., 80% of the total words which is 51,781 training samples. In contrast, this work has used only 3,000 annotated samples for training and 6,000 for the evaluation, in order to emulate low resource language with a limited amount of labelled data for training of supervised classifiers. Hence, for comparative analysis with the proposed model, a similar CNN architecture is used but with a limited labelled dataset retained for evaluation using supervised classification.

### Results and analysis

Classification accuracies are recorded over the proposed Auto-Encoder (AE) representation as well as traditional MFCC features for 10 iterations using different subsets of the labelled testing data. The proposed AE representation significantly outperforms the MFCC classifier with an average accuracy of 54.97% as compared to 45.71%. Additionally, Friedman’s test (Demšar, 2006) has also been used to analyse the statistical significance of the classification performance across at least 10 iterations, with the test suggesting that the results are significantly different with p and q values of 0.001565402 and 10, respectively. The non-parametric Friedman’s test is useful for comparison of classifier performance across input representations (Demšar, 2006).

For the 10 iterations, average precision and recall have also been recorded for both the proposed AE representation and traditional MFCC features. Precision refers to the ratio of true positives to the sum of true and false positives for each class whereas recall is a measure of true predictions divided by the sum of true positives and false negatives for that class. Table 1 lists averages for accuracy, recall, and precision across 10 iterations for the proposed AE representation and the traditional MFCC features, with ‘AE-P’ denoting the proposed positional constrained AE representation. Precision and recall are weighted averages of the metric scores across classes whilst accuracy is the percentage of totally correct predictions for each train and test iteration. It can be clearly seen that the averages of all three performance measures are significantly higher for the proposed AE representation.

**Table 1:** Average precision, recall, and accuracy across 10 iterations.

Average	MFCC	AE-P
Precision score	0.50	0.55
Recall score	0.45	0.54
Accuracy %	45.71	54.97

**Figure 5:**
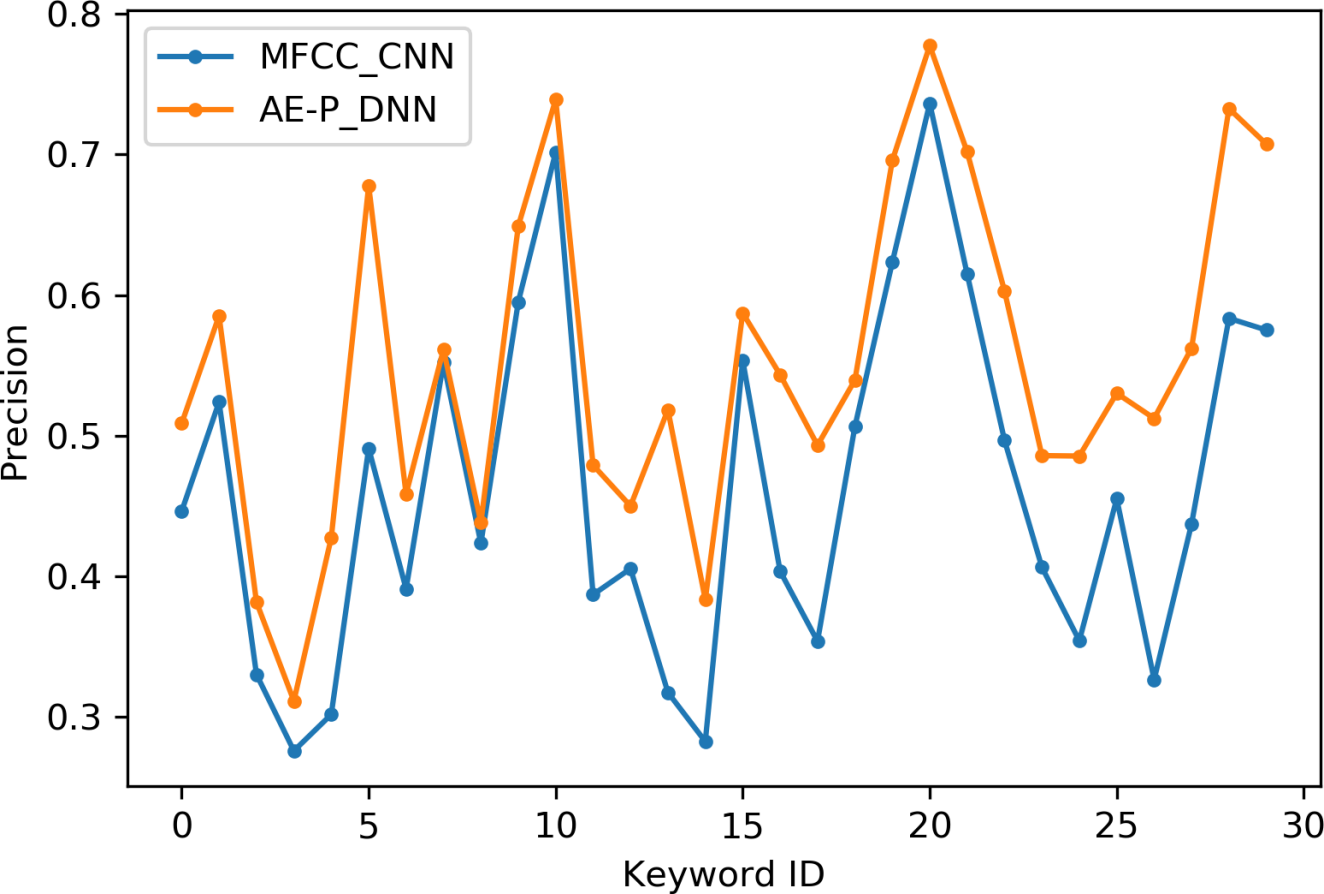
Precision for keywords. MFCC (blue), AE-P (orange).

**Figure 6:**
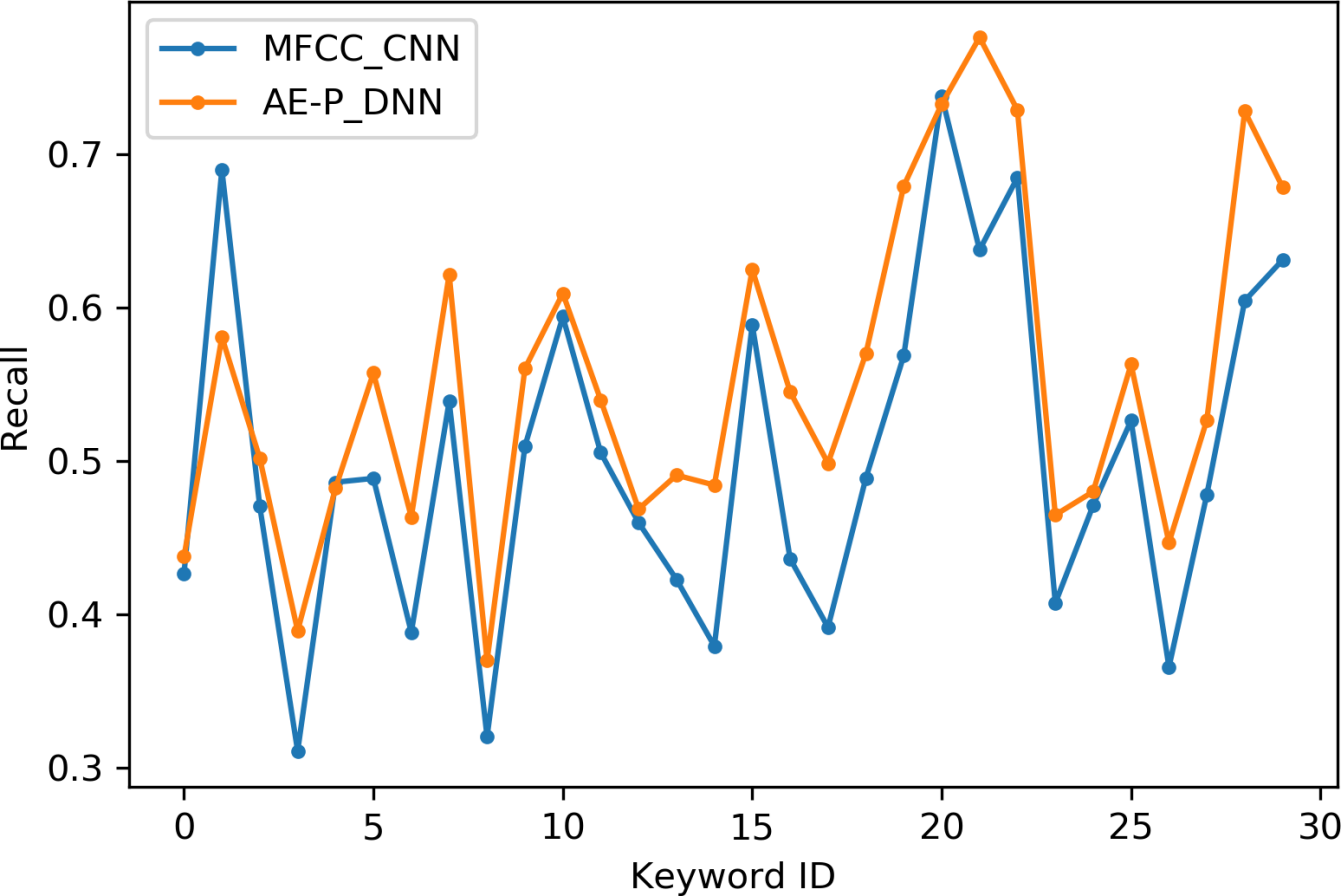
Recall for keywords. MFCC (blue), AE (orange).

**Figure 7:**
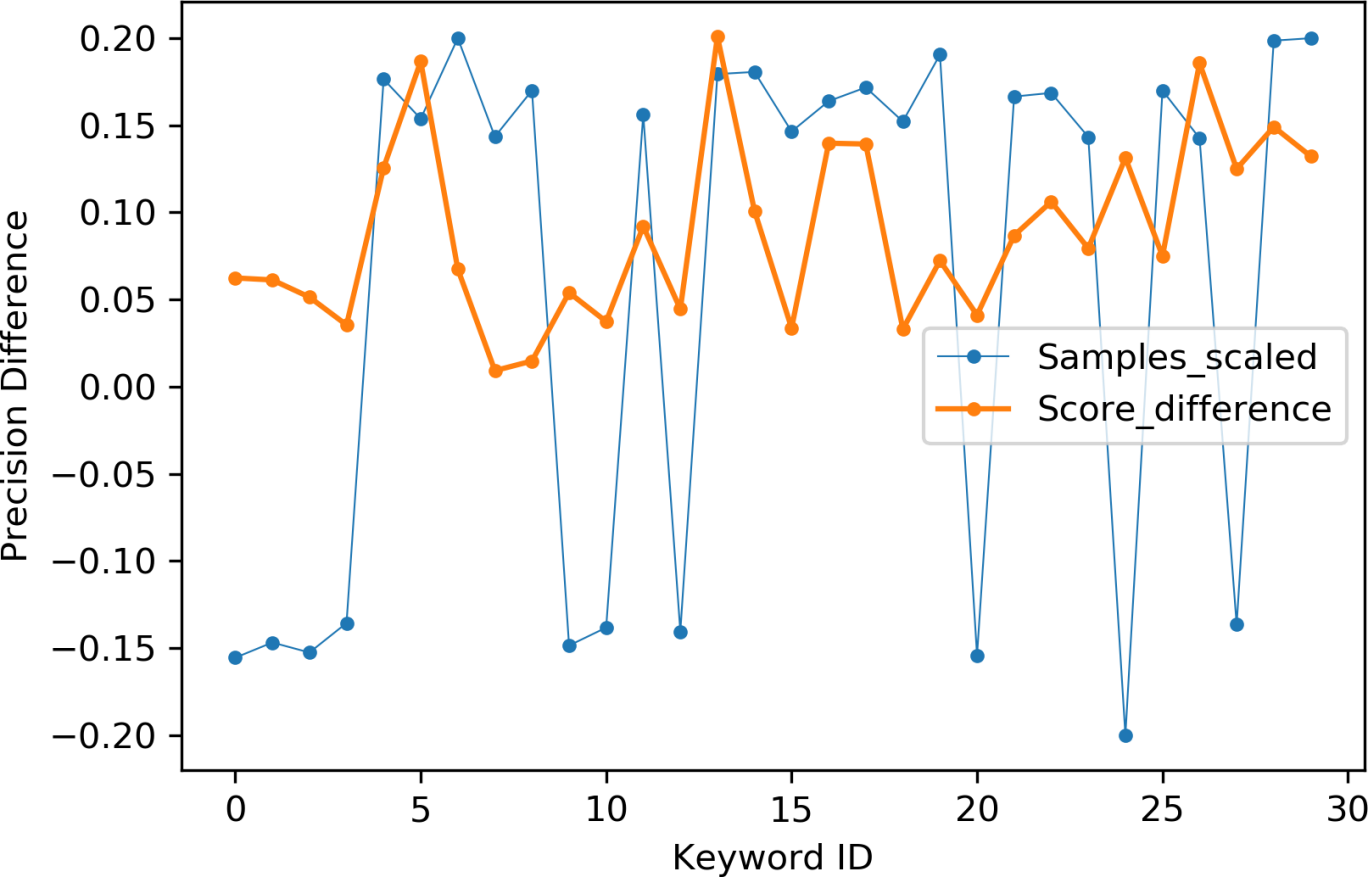
Precision difference between the proposed AE representation and MFCC. MFCC (blue), AE (orange).

**Figure 8:**
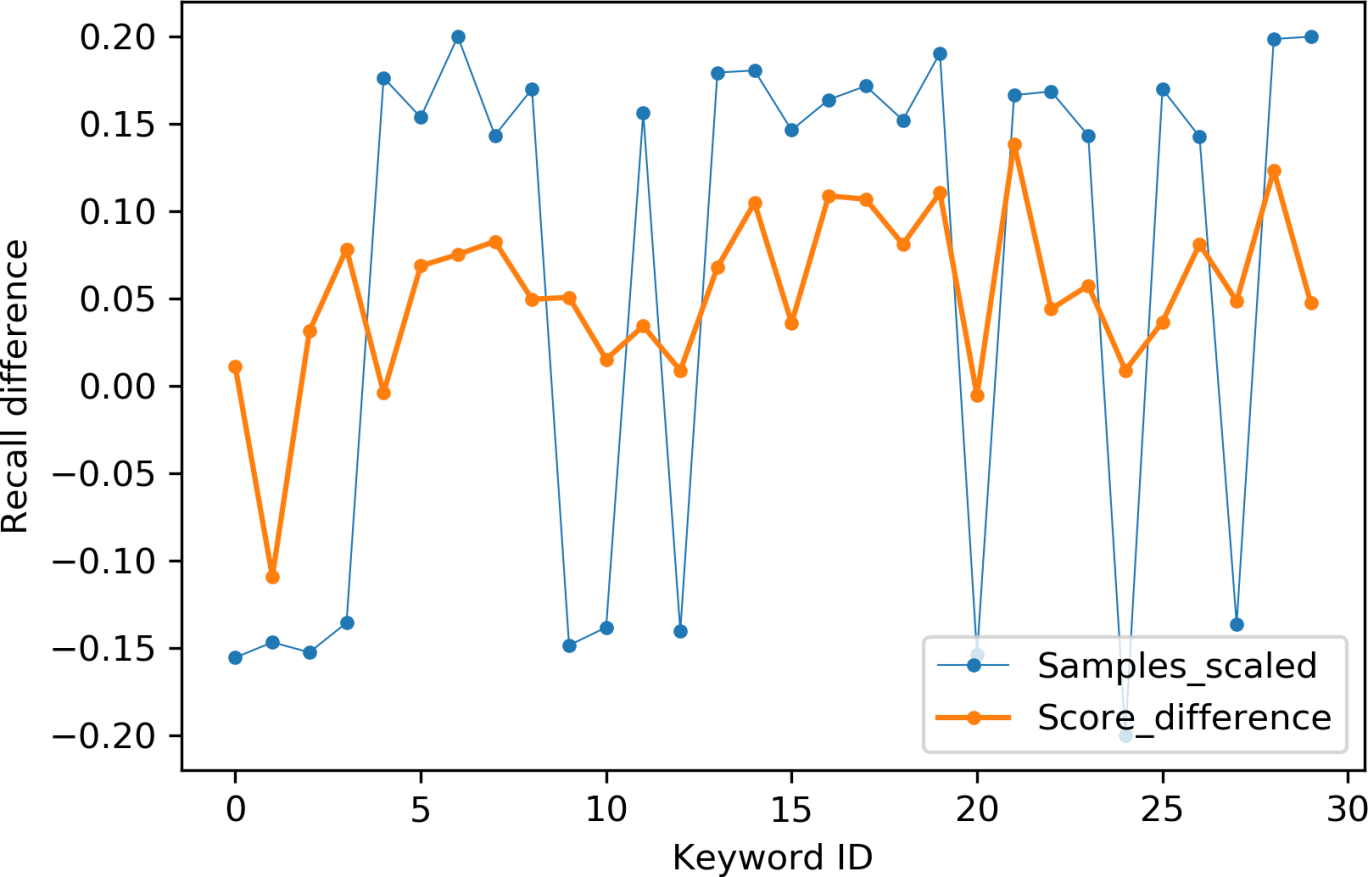
Recall difference between the proposed AE representation and MFCC. Sampels scaled (blue), score difference (orange).

Table 2 indicates the number of utterances for each keyword in the dataset along with keyword IDs, which have been assigned for convenient visualization on the axis labels in Figs. 5–8. Figures 5 and 6 illustrate precision and recall for individual keywords by the proposed AE representation and the traditional MFCC features. The category-wise analysis shows higher precision by the proposed AE representation than the MFCC features for all the keywords. In the case of recall, the score for the proposed AE representation is generally higher than traditional MFCC features with the exception of a few keywords. Figures 7 and 8 depict the differences in precision and recall scores between the proposed AE representation and the traditional MFCC features for all keywords along with the number of samples for each keyword. The number of samples is scaled to the range of precision–recall scores for visualization. As can be seen from Fig. 7, the proposed AE representation has considerably higher precision than the MFCC features for keywords: ‘eight’, ‘nine’, and ‘up’, whilst from Fig. 8, keywords: ‘six’ and ‘yes’, give higher recall score. On the other hand, the recall score for ‘bird’ is higher by the traditional MFCC features as compared to the proposed AE representation. Improvement in scores for the AE representation is generally higher for keywords with a higher number of utterances in the dataset, i.e., with more samples for the unsupervised training. Certain keywords have lower performance for both inputs due to their confusing sounds with other keywords. Keywords ‘Shiela’ and ‘six’ have both higher precision and recall for both the proposed AE representation and the MFCC features, whereas keyword ‘dog’ has low precision and recall for both. Precision and recall values for the keywords using MFCC and the AE-P representations have been tabulated in Table 3.

**Table 2:** ID and number of utterances for each keyword in the dataset.

Keyword	Keyword ID	Utterances
bed	0	2014
bird	1	2064
cat	2	2031
dog	3	2128
down	4	3917
eight	5	3787
five	6	4052
four	7	3728
go	8	3880
happy	9	2054
house	10	2113
left	11	3801
marvin	12	2100
nine	13	3934
no	14	3941
off	15	3745
on	16	3845
one	17	3890
right	18	3778
seven	19	3998
sheila	20	2022
six	21	3860
stop	22	3872
three	23	3727
tree	24	1759
two	25	3880
up	26	3723
wow	27	2123
yes	28	4044
zero	29	4052

**Table 3:** Precision and Recall for each keyword.

Keyword Name	Keyword ID	Precision MFCC	Recall MFCC	Precision AE-P	Recall AE-P
bed	0	4.46	4.27	5.09	4.38
bird	1	5.24	6.9	5.85	5.81
cat	2	3.3	4.7	3.82	5.02
dog	3	2.76	3.11	3.11	3.89
down	4	3.02	4.86	4.28	4.82
eight	5	4.91	4.89	6.78	5.57
five	6	3.91	3.88	4.59	4.64
four	7	5.52	5.39	5.61	6.22
go	8	4.24	3.2	4.38	3.7
happy	9	5.95	5.1	6.49	5.6
house	10	7.02	5.94	7.39	6.09
left	11	3.87	5.05	4.79	5.4
marvin	12	4.05	4.6	4.5	4.69
nine	13	3.17	4.23	5.18	4.91
no	14	2.83	3.79	3.83	4.84
off	15	5.54	5.89	5.87	6.25
on	16	4.04	4.36	5.43	5.45
one	17	3.54	3.92	4.93	4.99
right	18	5.07	4.89	5.4	5.7
seven	19	6.23	5.69	6.96	6.79
sheila	20	7.36	7.38	7.77	7.33
six	21	6.15	6.38	7.02	7.76
stop	22	4.97	6.85	6.03	7.29
three	23	4.06	4.07	4.86	4.65
tree	24	3.54	4.71	4.86	4.8
two	25	4.55	5.26	5.3	5.63
up	26	3.27	3.66	5.12	4.47
wow	27	4.37	4.78	5.62	5.27
yes	28	5.83	6.04	7.33	7.28
zero	29	5.75	6.31	7.07	6.79

**Figure 9:**
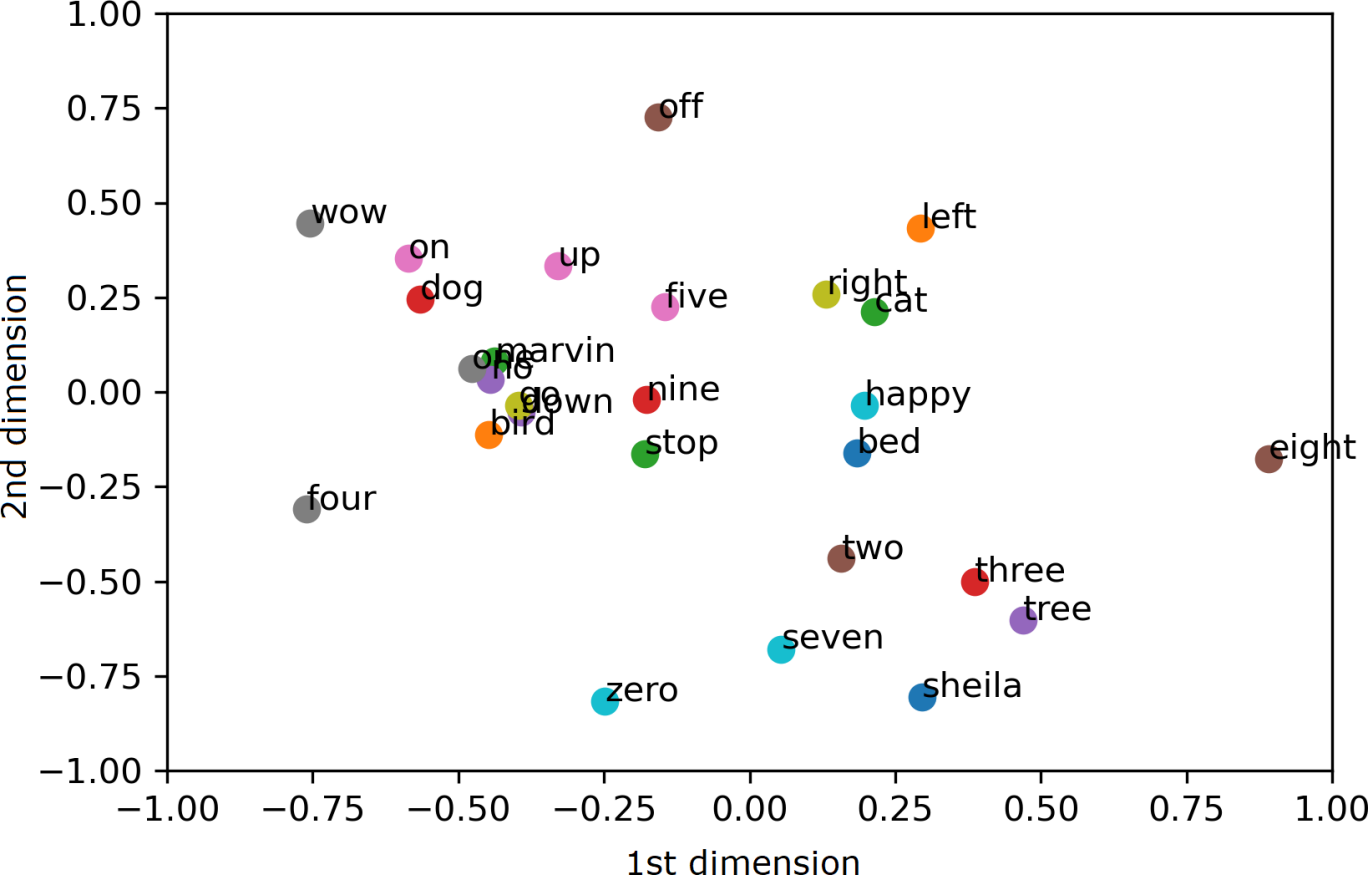
2D PCA projections for the proposed AE representation.

**Figure 10:**
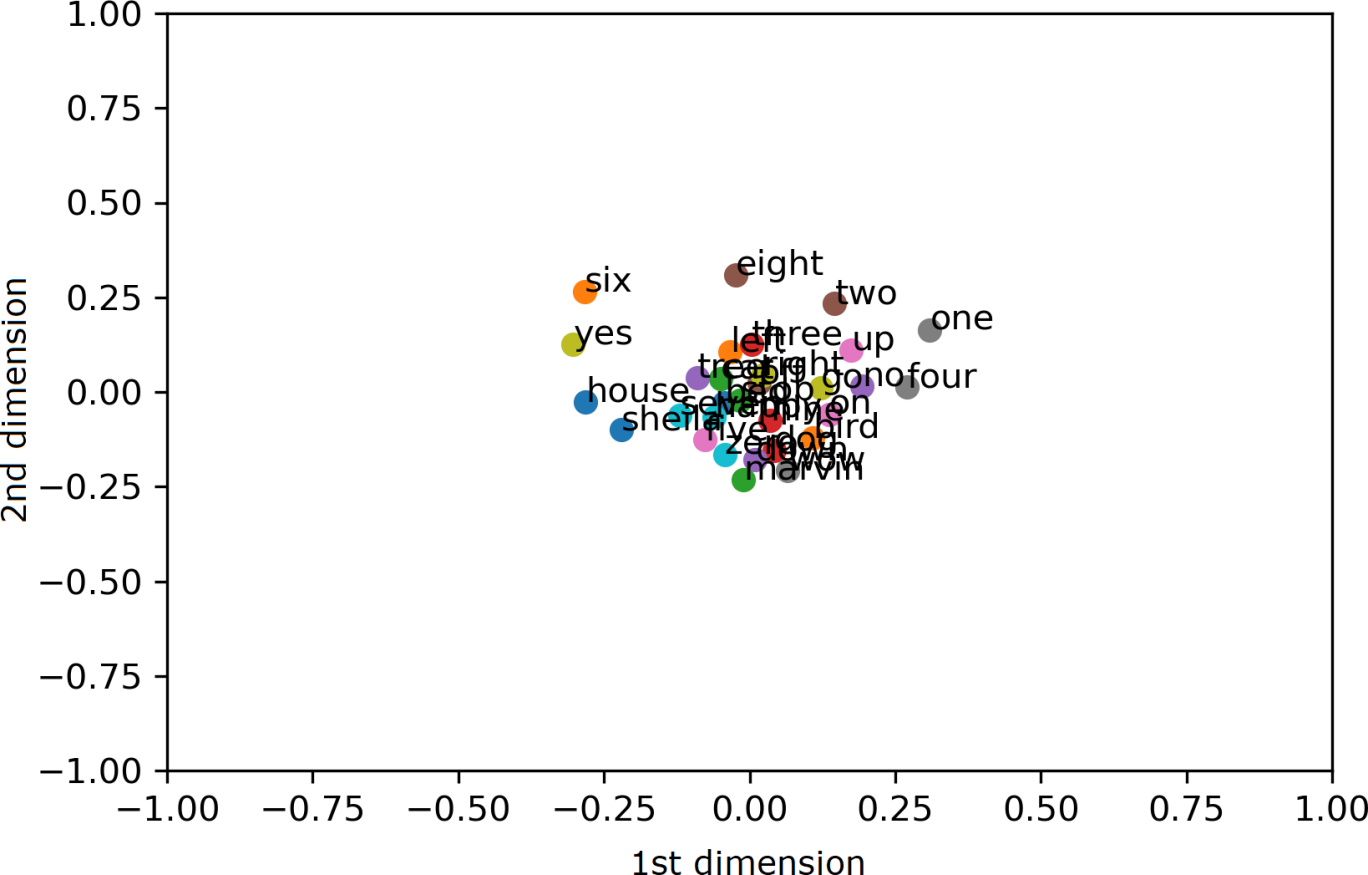
2D PCA projections for MFCC.

To visualize the segregation of words, both the AE representation and the traditional MFCC features are transformed into 2 dimensions (2D) using PCA projection. Scatter charts are plotted for the compressed 2D PCA projections for the proposed AE representation and the traditional MFCC features in Figs. 9 and 10, respectively. PCA is computed after normalization of both input features for comparison with the normalized representation. The 2 PCA components are displayed on the horizontal × and vertical y axes, with scales of both axes, fixed to 1 for visualization. Due to large amount of data, only the sample means of each keyword are plotted to highlight their separation; with the mean points labelled with the relevant keywords. Comparing Figs. 9 and 10, it can be seen that keywords are more separated in space with the proposed AE representation as compared to the traditional MFCC features.

**Figure 11:**
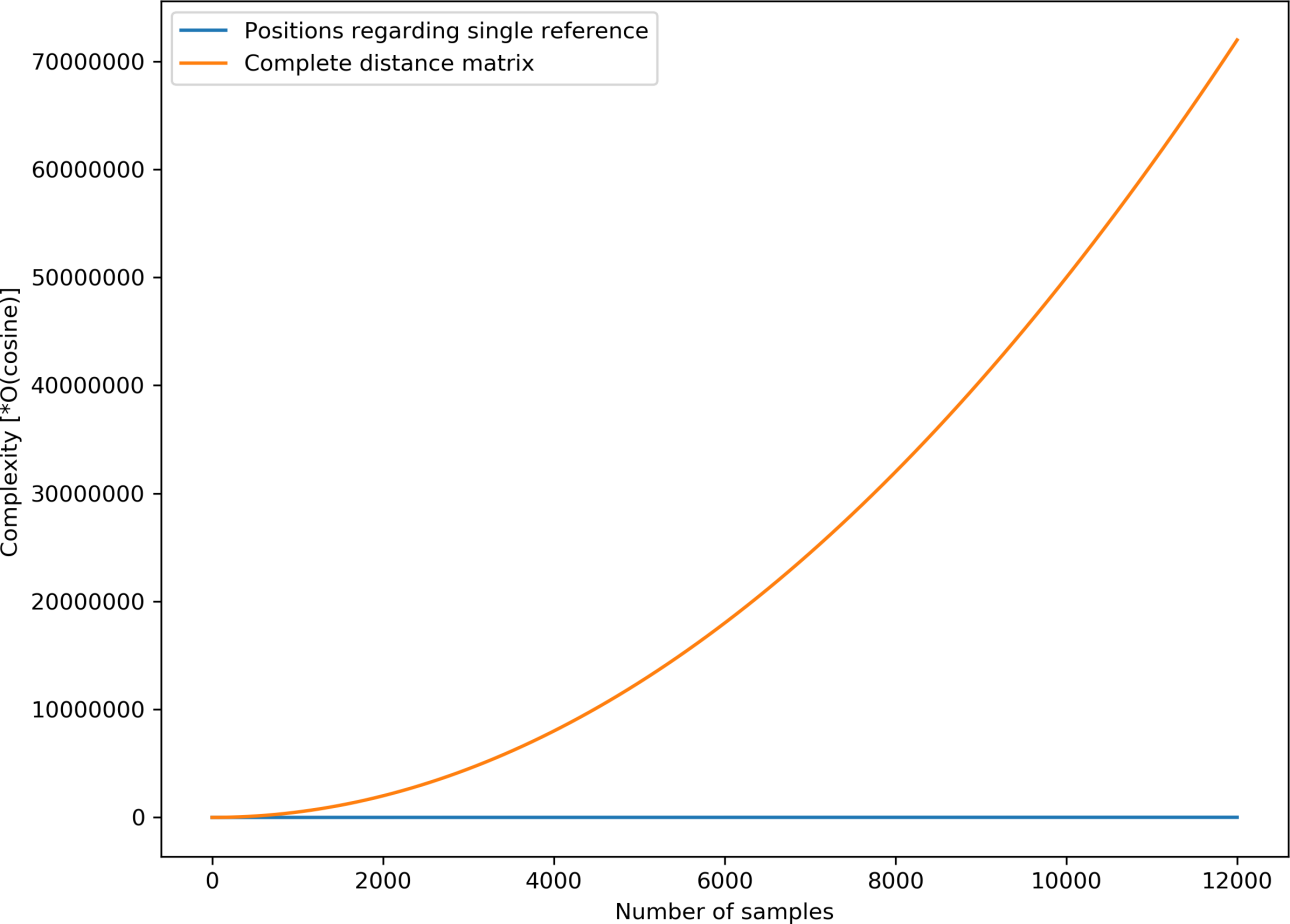
Computational complexities for complete distance matrix and the proposed position estimate.

Given that some speech representation learning models based on geometric distance rely on distance matrix computation which can be resource-intensive, it is appropriate to highlight the simplicity of the proposed AE representation. The complexity of distance matrix computation for *N* samples is given by O(Cosine)*N*(N-1)/2, where *O(Cosine)* is the complexity for cosine distance computation between a single pair of data points. For the proposed AE representation, the complexity is given by O(Cosine)*N. Given that *N* = 12,000 unlabelled samples are used for the unsupervised training, the complexity of computing the spatial positions for *12,000* unlabelled samples is simply 12,000*O(Cosine), which is significantly less than the complexity for calculating the complete distance matrix, i.e., (^12000^C_2_)*O(Cosine) = (7.2*10 ^7^)*O(Cosine) for an equal number of samples. Figure 11 highlights the stark differences in complexities between the two computations.

### Kadazan Language analysis

In order to analyse the model for a real low-resource language, a small dataset of spoken digits as keywords has been collected for the Kadazan Language. Kadazan language is an indigenous language spoken by the Kadazan people, a tribe living on the Western coast of the northern part of Borneo Island, which is the largest island in Asia. The language is included in the group of languages known as Dusun languages spoken in the Borneo Island. Whilst the Dusun languages have similarities with each other, they are significantly different from the Malay language, the official language in the region. The Kadazan language along with the other Dusun languages is facing extinction due to the fast declining linguistic experts and resources (Reid, 1997). The dataset collected for this research consists of the ten Kadazan digits spoken by 50 speakers, with the speech samples recorded using mobile handset microphones in noisy recording conditions. The ten digits for the Kadazan dialect are ‘Iso’, ’Duvo’, ’Tohu’, ’Apat’, ’Himo’, ’Onom’, ’Tuu’, ’Vahu’, ’Sizam’, and ’Opod’ (Humayun & Abas, 2021a).

Single channels have been selected and rescaled to a fixed duration of one second with a sampling rate of 16 kHz for processing. The pre-trained unsupervised model, trained with the unlabelled English dataset has been used to transform the rescaled Kadazan samples to their corresponding representations. The proposed representations as well as the flat MFCC vectors for the speech samples are then fed to similar supervised feed-forward KWS classifiers for comparative analysis on classification accuracies, with the tests performed by randomly selecting speakers for training and testing without overlapping. KWS classification accuracy has been recorded for both inputs across the number of speakers used for supervised training.

**Figure 12:**
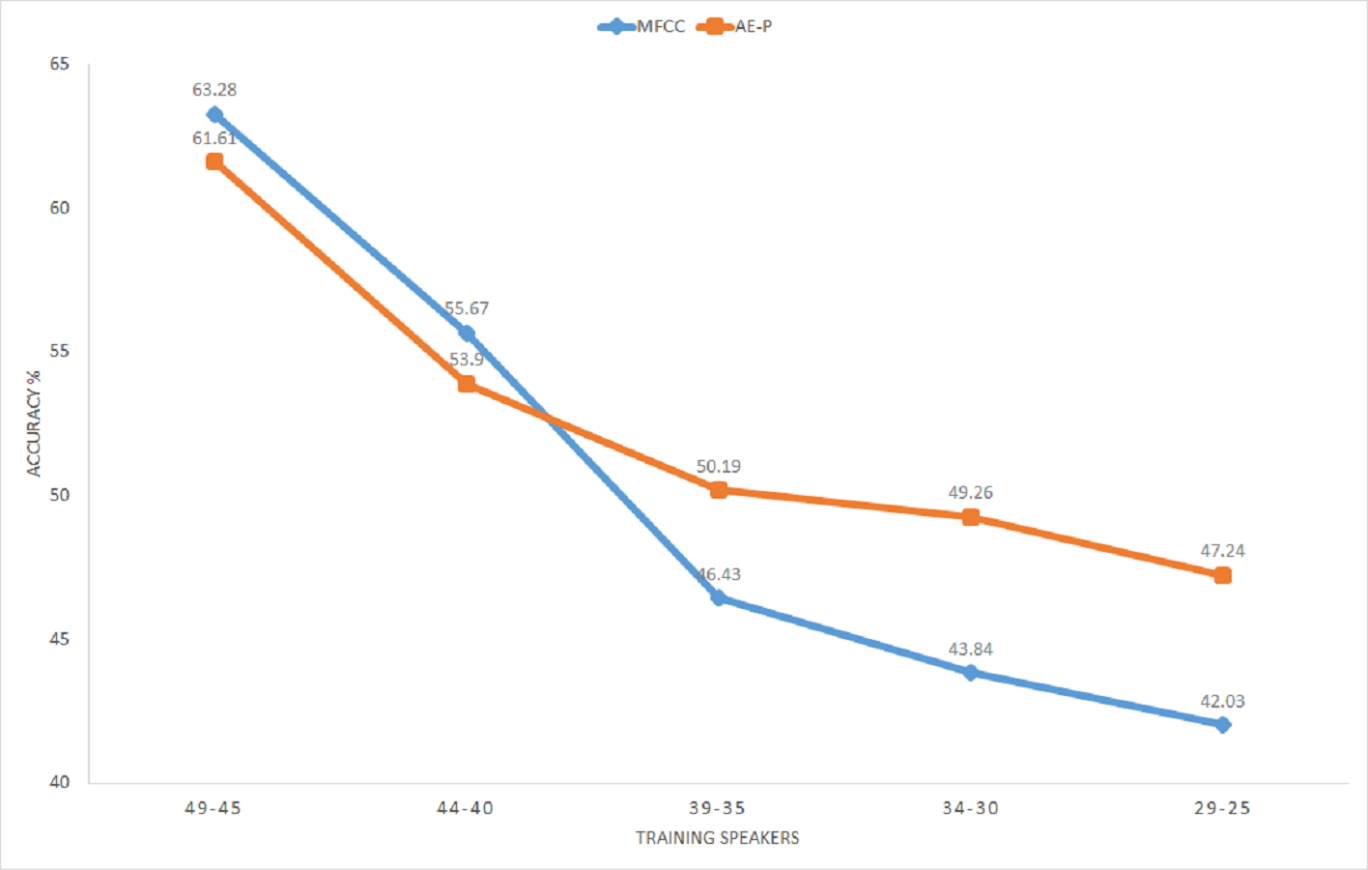
KWS accuracy for Kadazan digits across number of training speakers. MFCC (blue), AE (orange).

Figure 12 illustrates average classification accuracy with the range of speakers selected as training set for 10 iterations. The rest of the speakers from the total of 50 speakers in the dataset represent the test set for each iteration. It can be seen that MFCC vectors achieve better accuracy for a larger proportion of training speakers. On the other hand, accuracy using the proposed representation improves with decreasing number of training speakers, surpassing MFCC with 35 training speakers or less. The representation achieves around 5% better average accuracy for the range of 34 to 25 training speakers. Better accuracy by the proposed representation for a smaller proportion of training speakers substantiates its relevance for limited annotation settings. The result further shows that the unsupervised model trained with the English dataset performs well for extracting features from the Kadazan speech, which confirms the ability of the proposed model to learn cross-lingual features.

## Conclusions

The paper proposes a geometric position based multitasking auto-encoder for unsupervised speech representation learning with geometric position of points described by cosine distance from a common reference. This geometric position is then added as a secondary regression task for the auto-encoder. Unlike other speech representation learnings, the proposed model does not require random sampling nor complete distance matrix computation for the preservation of the spatial structure from the input feature space. Subsequently, the proposed model has been evaluated by using its learnt representation for KWS, where it has been shown that the proposed AE representation achieves significantly higher accuracy (around 9%) as compared to the traditional MFCC features with limited labelled data. Visual inspection has also confirmed that the proposed representation is able to separate selected keywords in space. Significant improvement in accuracy by the unsupervised representation confirms the usefulness of the proposed model for classification problems with limited labelled data. Similar experiments have been conducted for a small dataset of keywords in Kadazan language for evaluation using actual low-resource language. KWS results for Kadazan dataset with varying size of labelled training set confirms that the proposed representation gives better performance than MFCC as the labelled set size gets smaller.

Comparison of classification scores by similar supervised classifiers using the MFCC vectors and the proposed unsupervised representation illustrates the ability of the proposed representation to learn critical information from unlabelled data. Furthermore, the monotonically increasing improvement in accuracy by the proposed representation in comparison to MFCC with decreasing labelled set establishes its utility for low-resource settings. Finally, the effectiveness of the unsupervised model trained and tested with different languages confirms its capability of extracting useful features irrespective of the spoken language. This generalization of the model across languages makes it pertinent for low-resource languages.

The obtained results have answered the posed research questions in this paper. It is evident from the results that the proposed geometry-based auto-encoder can learn to effectively extract speech features which are useful for the keyword spotting task, by training the model using unlabeled speech. Better performance in accuracy is achievable by using the proposed method to extract features, as compared to using the traditional MFFC features. Further, the applicability of the proposed method has been illustrated on a real low-resource Kadazan language.

However, it is worthy to note that the evaluation has been performed on a small footprint KWS task for low vocabulary isolated words and as such, the performance of the representation needs to be further evaluated for Large Vocabulary Continuous Speech Recognition (LVCSR). Size of the Kadazan dataset is also small in terms of spoken keywords as well as the speakers. Moreover, the number of reference anchors and their locations need to be further explored for the computation of spatial positions, on top of testing the model on paralinguistic speech processing tasks. Finally, input data augmentation can be employed in the auto-encoder training for learning the representation to make it more invariant to irrelevant speech and environmental characteristics.

##  Supplemental Information

10.7717/peerj-cs.650/supp-1 Supplemental Information 1 Spatial-AE Python code

## Additional Information and Declarations

### Data Availability

The following information was supplied regarding data availability: The code file for our tests are available in the Supplementary File. The dataset used for analysis is available from TensorFlow: https://www.tensorflow.org/datasets/catalog/speech_commands.
